# Genetically engineered rat gliomas: PDGF-driven tumor initiation and progression in *tv-a* transgenic rats recreate key features of human brain cancer

**DOI:** 10.1371/journal.pone.0174557

**Published:** 2017-03-30

**Authors:** Nina P. Connolly, Jesse A. Stokum, Craig S. Schneider, Tatsuya Ozawa, Su Xu, Rebeca Galisteo, Rudolph J. Castellani, Anthony J. Kim, J. Marc Simard, Jeffrey A. Winkles, Eric C. Holland, Graeme F. Woodworth

**Affiliations:** 1 Department of Neurosurgery, University of Maryland School of Medicine, Baltimore, Maryland, United States of America; 2 Marlene and Stewart Greenebaum Comprehensive Cancer Center, University of Maryland School of Medicine, Baltimore, Maryland, United States of America; 3 Fred Hutchinson Cancer Research Center, University of Washington, Seattle, Washington, United States of America; 4 Department of Diagnostic Radiology and Nuclear Medicine, University of Maryland School of Medicine, Baltimore, Maryland, United States of America; 5 Department of Surgery, University of Maryland School of Medicine, Baltimore, Maryland, United States of America; 6 Department of Pathology, University of Maryland School of Medicine, Baltimore, Maryland, United States of America; 7 Department of Physiology, University of Maryland School of Medicine, Baltimore, Maryland, United States of America; University of Florida, UNITED STATES

## Abstract

Previously rodent preclinical research in gliomas frequently involved implantation of cell lines such as C6 and 9L into the rat brain. More recently, mouse models have taken over, the genetic manipulability of the mouse allowing the creation of genetically accurate models outweighed the disadvantage of its smaller brain size that limited time allowed for tumor progression. Here we illustrate a method that allows glioma formation in the rat using the replication competent avian-like sarcoma (RCAS) virus / tumor virus receptor-A (*tv-a)* transgenic system of post-natal cell type-specific gene transfer. The RCAS/*tv-a* model has emerged as a particularly versatile and accurate modeling technology by enabling spatial, temporal, and cell type-specific control of individual gene transformations and providing *de novo* formed glial tumors with distinct molecular subtypes mirroring human GBM. *Nestin* promoter-driven *tv-a (Ntv-a)* transgenic Sprague-Dawley rat founder lines were created and RCAS PDGFA and p53 shRNA constructs were used to initiate intracranial brain tumor formation. Tumor formation and progression were confirmed and visualized by magnetic resonance imaging (MRI) and spectroscopy. The tumors were analyzed using histopathological and immunofluorescent techniques. All experimental animals developed large, heterogeneous brain tumors that closely resembled human GBM. Median survival was 92 days from tumor initiation and 62 days from the first point of tumor visualization on MRI. Each tumor-bearing animal showed time dependent evidence of malignant progression to high-grade glioma by MRI and neurological examination. Post-mortem tumor analysis demonstrated the presence of several key characteristics of human GBM, including high levels of tumor cell proliferation, pseudopalisading necrosis, microvascular proliferation, invasion of tumor cells into surrounding tissues, peri-tumoral reactive astrogliosis, lymphocyte infiltration, presence of numerous tumor-associated microglia- and bone marrow-derived macrophages, and the formation of stem-like cell niches within the tumor. This transgenic rat model may enable detailed interspecies comparisons of fundamental cancer pathways and clinically relevant experimental imaging procedures and interventions that are limited by the smaller size of the mouse brain.

## Introduction

Glioblastoma (GBM), the most common and deadly primary brain cancer, has a dire prognosis [1, 2]. Even with aggressive treatments, consisting of maximal safe surgical resection with adjuvant chemoradiation therapy, the tumors almost uniformly recur and median survival is less than 18 months [3]. Although there are several new therapeutic approaches in development, including engineered T-cells, immune checkpoint inhibitors, and precision medicines, meaningful change to the current clinical protocols has not occurred in more than 10 years.

Emerging information related to GBM disease mechanisms has revealed the importance of tumor size, heterogeneity, and specific molecular features in efforts to accurately model and confront this complex disease. While gliomas have been traditionally characterized by histologic grade, where GBM (grade IV glioma) represents the most malignant form, molecular classification schemes have been developed recently based on specific gene mutation and expression patterns [1, 2, 4, 5]. These molecular features are leading to new insights into the tumor biology, treatment choice and likelihood of response, and patient prognosis. Yet, single cell and regional analyses of individual tumors have also revealed that high-grade glial tumors consist of heterogeneous mixtures of cells representing multiple molecular subtypes, components of which may evolve from a common proneural-like precursor [1, 2, 6, 7]. As it becomes more clear that GBM molecular features and intratumoral heterogeneity play important roles in predicting tumor evolution, treatment responses, and patient prognosis, animal models that recapitulate large, pleomorphic tumors and enable clinical imaging and diverse treatment paradigms will likely have significant value in future pre-clinical studies.

Numerous animal models (rodents, rabbits, monkeys, dogs) have been studied over the last 50 years to study GBM in various contexts, including spontaneous canine tumors, mutagen-induced transplantable cell lines grown in syngeneic host animals, patient-derived xenografts (PDXs) grown in immune-deficient host animals, and more recently, genetically engineered (GE) animals that enable spontaneous and inducible tumor formation [8, 9]. Each of these models offers pros and cons regarding predictive accuracy, experimental design and reproducibility. Mutagen-induced cell lines are often immortalized and can be passaged and manipulated *in vitro* over long periods of time, then transplanted into syngeneic hosts in a controlled, reproducible fashion. These models have been developed in mice, rats, and rabbits, and have been used in a wide range of pre-clinical studies, despite uncertain relevance to human disease [10]. PDXs offer the direct study of human tumors; however, *in vitro* culturing and manipulation can significantly alter the cell biology and *in vivo* transplantation requires an immunocompromised host environment to prevent rejection [11–13]. Spontaneous glioma in canines may have the most biological overlap with human brain cancer but high cost, relatively few experimental reagents, and significant ethical considerations limit the broad application of this model in pre-clinical studies. GE models have been hindered by the need for complex breeding and genotyping, low or variable penetrance of the tumor phenotype, and lack of tumor complexity [8, 9, 14]. Yet, GE models enable *de novo* tumor formation in immunocompetent animals and numerous experimental systems can be used to engineer many of the biological alterations found in human cancer. Thus, *de novo* tumor formation in the host environment significantly differentiates GE from cell transplant-based models, and creates the opportunity to study tumor initiation, progression, and treatments where the disease microenvironment also evolves in the context of the neoplastic processes.

A powerful and versatile technology for GE modeling utilizes the replication competent avian-like sarcoma (RCAS) virus and its avian tumor virus A (*tv-a*) cell surface receptor [15]. In this GE system, the *tv-a* gene is stably inserted into a host and controlled by a cell type- or tissue-specific promoter such as the *nestin* promoter, which is activated in neural and glial progenitor cells—the cells implicated as brain tumor initiating cells (BTICs) [16, 17]. The expression of the *tv-a* receptor on BTICs serves as a port of entry for *tv-a* specific RCAS viruses engineered to alter the expression of genes linked to the development of human cancer. Indeed, the forced expression of oncogenes (e.g. RAS, PDGF) and/or functional loss of tumor suppressor genes (e.g. p53, PTEN, NF1) using the RCAS/ *tv-a* system leads to distinct glioma subtypes in *tv-a* transgenic mice [7, 18]. Studies using the *tv-a* transgenic mice have led to numerous biological and translational discoveries related to brain cancer [18–21]. Accordingly, we were interested in studying brain cancer in *tv-a* transgenic rats to enable the generation of larger, heterogeneous gliomas that can be visualized over time using MRI and offer the potential for a broad range of therapeutic delivery and comparative tumor biology studies.

In this study, we established stable, *nestin* promoter-driven *tv-a* (*Ntv-a*) transgenic rats. We then introduced the gene transformations previously shown to induce proneural-like brain tumors in mice, and characterized tumor initiation and progression using MRI and MR spectroscopy (MRS). Detailed post-mortem histopathological analysis performed using immunohistochemistry and immunofluorescence microscopy suggested high similarity to the human disease.

## Materials and methods

### Generation and characterization of *tv-a* transgenic Sprague-Dawley rats

The *Ntv-a* transgene was created by replacing the *lacZ* gene in the *nestin* (NES) 1689/*lacZ* plasmid with cDNA encoding *tv-a* (*Ntv-a*), as previously described [22]. This construct was used to established transgenic Sprague-Dawley (SD) rat founders (Transposagen Biopharmaceuticals Inc., Lexington, Kentucky). Briefly, the *Ntv-a* cDNA was cloned into a plasmid with Sleeping Beauty (SB) terminal repeats, making a SB transgene construct. This construct was injected along with the SB transposase into SD rat embryos which then implanted into fertile female SD rats. The offspring were bred with wild-type Sprague-Dawley rats to produce the first generation (F_1_) progeny. These F_1_ animals were analyzed for transgene expression using standard PCR-based methods [23] and expression was confirmed using immunohistochemical techniques. Animals were genotyped to confirm homozygous *tv-a* gene integration using DNA extracted from tail samples and PCR using TV-A primers (forward primer sequence 5’-CTCACCACGGAACCTGACTC-3’ and reverse primer sequence 5’-ATTCAAAGCCTCCAGCAAAA-3'; 58°C annealing temperature; 32 cycles). Quantitative PCR (qPCR) was used to determine the copy number for all offspring, primarily for breeding. Primers for qPCR were designed using a software program available on the Integrated DNA Technologies (IDT, Coralville, Iowa) website. The primer sequences were: TV-A (Forward 5’-TGCTCCTGGACGTGCTCT-3’; Reverse 5’-GCAGTGATCAGCATCCACAT-3’) and Actin (Forward 5’-AAGTCCCTCACCCTCCCAAAAG-3’; Reverse 5’-AAGCAATGCTGTCACCTTCCC-3’). qPCR was performed with SYBR^®^ Green PCR Master Mix (ThermoFisher, Waltham, MA) using BioRad CFX96 Detection instrument and software (Hercules, CA). For each reaction, 2 μl of cDNA (100 ng) was used per 25 μl reaction with the following cycling conditions: an initial denaturation at 95° for 5 min followed by 40 cycles of 95°, 55°, then 72° each for 30 s. A melting curve step was added to include 96° for 10 s followed by 65° for 5 min. All samples were run in triplicate and normalized to actin. The mean Cq value of the triplicate samples was used for copy number determination. The copy number of each animal was determined using a serial dilution of the *nestin* plasmid (Transposagen) ranging from 300 to 300,000 copies. The dilution series was run using the same qPCR protocol as above. Once mean Cq values were obtained, a linear equation based on the standard curve was determined. Cq values of the cDNA samples were calculated using the standard curve equation that yielded the log copy number for each animal. Those animals with the highest percentage of homozygous *Ntv-a* positive siblings were analyzed for *Ntv-a* total copy number [23]. These high copy number siblings were then interbred to establish the initial *Ntv-a* transgenic colony. The genotyping and copy number analysis were performed with each subsequent generation, after which the high copy number males were back-crossed with wild-type SD females to maintain the health of the colony.

### Cell culture and transfection

The RCAS plasmids encoding firefly luciferase (FLuc) and platelet-derived growth factor-A (PDGFA) were described previously [24]. The rat p53 short hairpin RNA (shRNA) plasmid (target sequence, 5’-GTCAGGGACAGCCAAGTCTGT-3') [25]) was constructed by Genecopia (Rockville, Maryland) and inserted along with the mCherry fluorescent reporter protein gene. All plasmids were amplified and purified by Aldevron Inc. (Fargo, North Dakota).

DF-1 immortalized chicken fibroblasts (ATCC^®^ CRL-12203^™^, American Type Culture Collection, Rockville, MD) were used to generate RCAS virions as described previously [26, 27]. Separate flasks of DF-1 cells were transfected with each RCAS plasmid using FuGene 6 transfection reagent (Roche Diagnostics, Indianapolis, IN) following the manufacturer’s protocol. Cells were incubated for 48 h and successful transfection with the p53 shRNA/mCherry plasmid was confirmed by fluorescence microscopy. Transfection medium was subsequently removed and the cells were passaged three times and then frozen. These low passage (4 passages or less) DF-1 stocks were used for all experiments.

### In vivo gene transfer and tumor formation

All animal experiments were conducted in accordance with protocols approved by the University of Maryland School of Medicine Institutional Animal Care and Use Committee (IACUC) and followed NIH guidelines for animal welfare. DF-1 cells transfected with specific RCAS plasmids were harvested using Trypsin/EDTA solution (Corning, Tewksbury, MA), and then washed and resuspended in PBS. To check the function of the *Ntv-a* transgene *in vivo*, the RCAS-FLuc producing DF-1 cells were injected (5x10^5^ cells in 5 μl) into the subventricular zone and basal ganglia of three animals using standard stereotactic techniques and coordinates [28, 29] on postnatal day (PND) 10. RCAS-PDGFA producing DF-1 cells were also injected in order to stimulate prolonged FLuc expression. At 24 h post-injection, animals were injected i.p. with 10 μl/g body weight of D-Luciferin monopotassium salt (5 mg/ml solution, Thermo Scientific) 10 min before imaging. They were then imaged (1 min exposure) using the Xenogen IVIS Imaging System (PerkinElmer, Waltham, MA) and whole body images were acquired [30, 31]. Animals were imaged every 24 h for a week and then every 48 h until the bioluminescence signal was lost. MRI and MRS were used to follow tumor formation. In subsequent experiments, DF1 cells producing RCAS-PDGFA and RCAS-shp53 (expressing an shRNA to p53) were mixed 1:1 (5x10^5^ cells in 5 μl) and co-injected into PND10 rats as above. Animals were monitored for tumor growth using MRI and MRS.

### Magnetic resonance imaging and spectroscopy

All MRI scans were performed using a Bruker BioSpec 70/30USR Avance III 7T horizontal bore MR scanner (Bruker Biospin MRI GmbH, Germany) equipped with a BGA12S gradient system and interfaced to a Bruker Paravision 5.1 console. A Bruker four-element ^1^H surface coil array was used as the receiver and a Bruker 72 mm linear-volume coil as the transmitter. Rats were placed inside the magnet using a body holder. Rats were under 1–2% isoflurane anesthesia and 1 L/min oxygen administration during the imaging. A MR compatible small-animal monitoring system was used to monitor the rat’s respiration rate and body temperature. Body temperature was maintained at 35–37°C using warm water circulation.

T_2_-weighted coronal MR images covering the entire brain were obtained using a 2D rapid acquisition with relaxation enhancement (RARE) sequence with repetition time/effective echo time (TR/TE_eff_) = 4500/45 ms, echo train length = 4, field of view (FOV) = 3.5 x 3.5 cm^2^, matrix size = 256 x 256, slice thickness = 0.5 mm, number of averages = 4. For a group of selected animals, pre- and post-gadobenate dimeglumine (Gd-BOPTA, 0.2 mL/kg) enhanced T_1_-weighted coronal images were performed using the same RARE sequence with TR/TE = 342/9 ms, echo train length = 1, slice thickness = 1.0 mm. The rest parameters were the same as T_2_-weighted images. Gd-BOPTA was injected through catheterized tail vein. A short-TE PRESS (point-RESolved Spectroscopy) pulse sequence [32] (TR/TE = 2500/10 ms) was used for MRS data acquisition from both the tumor region and the contralateral cerebral hemisphere, respectively. The voxel size of each rat was varied to fit to the size of the tumor. The acquisition time for each voxel was fixed to 25 min. The contralateral voxel size was kept the same as the tumor voxel. Unsuppressed water signal from each of the prescribed voxels was obtained to serve as a reference for determining the specific biochemical concentrations. LCModel package [33] was used for quantification of the MRS data. The reliability of the major biochemical entities was estimated in the Cramér-Rao lower bounds (CRLB) from the LC Model analysis [32, 33].

MRS enables characterization of the evolution of the neoplastic process, similar to the use of MRS in clinical practice. Commonly used methods analyze the cell membrane component choline (Cho), the marker of energy stores—creatine and phosphocreatine (Cr), and the neuronal marker N-acetylaspartate (NAA), as well as other chemical entities within the brain tissue. Generally, Cho/Cr ratio >1.5 represents increased cellular turnover and metabolic activity, suggestive of neoplasia. As non-neuronal lesions expand within the brain, neurons are displaced and the NAA peak decreases. Imaging studies were conducted at 30–40 days following the initial injection and/or when the animals began to show behavioral and neurological symptoms. Images were collected and reviewed with a neuroradiologist (S.X.) to assess the tumor regions and associated characteristics.

### Brain tissue collection

The experimental endpoint for the timing of euthanasia was determined based on IACUC guidelines, which included imaging evidence of large tumor with mass effect, neurological symptoms, >10% weight loss, and poor grooming. Animals were euthanized with induction of general anesthesia followed by exsanguination using transcardiac perfusion of cold phosphate buffered saline. The brain was rapidly extracted and sectioned into 2-mm thick sections. Tumor-containing sections were fixed in 4% formalin for 24 h and transferred to 70% ethanol for immunohistochemistry or 30% sucrose for immunofluorescence before embedding in paraffin or OCT compound, respectively.

### Histopathological analyses

Fixed tissues were mounted in paraffin blocks using the Leica EG 1160 embedding center (Leica Microsystems, Buffalo Grove, IL) and then sectioned in 5 μm slices oriented in the coronal plane. Sections were stained and examined using standard histopathological techniques (hematoxylin & eosin, immunohistochemistry) and reviewed with a neuropathologist (R.C.). Antigen retrieval was performed using either Bond Epitope Retrieval Solution 1 (pH ~6) or Bond Epitope Retrieval Solution 2 (pH ~9) (Leica Microsystems) at 99–100°C for 20–30 min. IHC staining was performed on a Leica BOND-III^™^ autostainer (Leica Microsystems) and peroxidase/DAB Bond^™^ Polymer Refine Detection System (Leica Microsystems) was used for visualization. Primary monoclonal antibodies used for IHC detected the following proteins: TV-A (1:200; provided by Dr. Andrew Leavitt, University of California, San Francisco), Ki-67 (1:100; 275R-18, Sigma-Aldrich, Saint Louis, MO), p53 (1:300; PA0057, Leica Microsystems), glial fibrillary acid protein (GFAP) (1:200; PA0026, Leica Microsystems), oligodendrocyte lineage transcription factor 2 (OLIG2) (1:200; 387M-18, Sigma-Aldrich), Nestin (1:200; 14-5843-82 eBioscience, San Diego, CA), CD44 (1:100; ab157107, Abcam, Cambridge, MA), smooth muscle action [SMA] (1:100; sc-32251, Santa Cruz Biotechnology, Dallas, TX), PDGFA (1:200; sc-128; Santa Cruz Biotechnology), and mCherry (1:800; 600-401-P16, Rockland Antibodies, Limerick, PA).

### Immunofluorescence microscopy

Coronal cryosections (12 μm) of rat brains were obtained from the center of the anterior-posterior extent of the tumor. Tissues were blocked for 1 h in 2% donkey serum with 0.2% Triton X-100 and labeled overnight at 4°C with primary antibodies against GFAP (1:500; C9205, Sigma-Aldrich), ED-1 (1:200; MAB 1435, Millipore, Billerica, MA), RECA-1 (1:200; MA1-81510, Thermo Fisher Scientific Inc.), HIF-1 (1:200; sc-12542, Santa Cruz Biotechnology, Inc.), CD44 (1:200; ab157107, Abcam), Olig2 (1:100; ab109186, Abcam), CD3 (1:200; ab5690, Abcam), CD8a (1:200; ab33786, Abcam), CD4 (1:200; sc-1573, Santa Cruz Biotechnology, Inc, CD49d (1:200; ab22858, Abcam), P2Y12 (1:200; AS-55043, AnaSpec, Fremont, CA) or IBA1 (1:100; ab5076, Abcam). Alexa Fluor 500- or fluorescein isothiocyanate-conjugated secondary antibodies were applied, and tissues were coverslipped with ProLong Gold antifade reagent (P36930; Thermo Fisher Scientific Inc.). Specific labeling was confirmed by omission of primary antibody. Immunolabeled tissues were visualized with epifluorescence microscopy (Nikon Eclipse 90i; Nikon Instruments Inc., Melville, NY).

## Results

**Fig 1:**
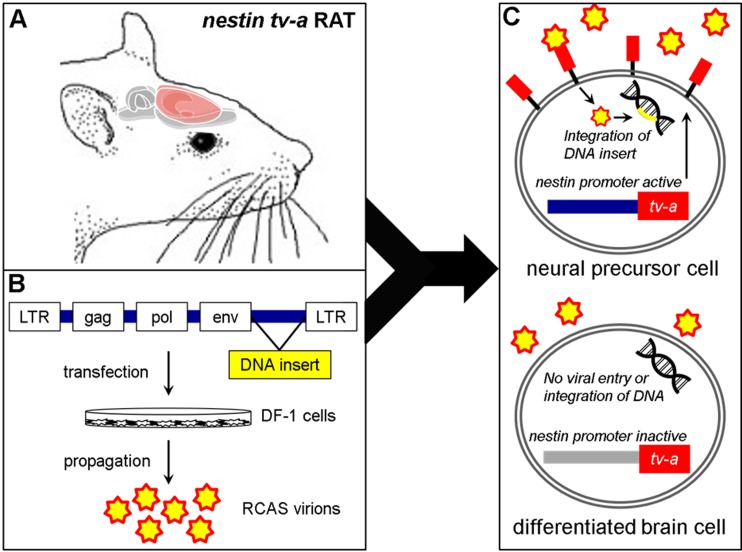
Schematic depiction of the RCAS-*Ntv-a* rat glioma model. **(A)** Transgenic Sprague-Dawley rats were created using site-specific transposase technology [34] with the *tv-a* gene under control of the *nestin* promoter. **(B)** Chicken fibroblast DF1 cells are transfected with RCAS constructs (e.g. PDGFA and p53 shRNA) that propagate and release RCAS virions into the culture media **(C)** DF1 cells are injected into the transgenic rats and the RCAS virions bind to the *tv-a* receptor expressed on neural precursor cells in which the nestin gene promoter is active. Once inside the cell, the DNA insert is integrated to produce stable gene expression changes leading to tumor formation. RCAS virions do not enter differentiated brain cells where the nestin promoter is inactive. (adapted from Ahronian *et al*., 2014 [35]).

An overview of the rat RCAS-*Ntv-a* model of glioma initiation and progression is shown in Fig 1. First, an *Ntv-a* transgenic rat colony was established, characterized for transgene copy number, and tested to confirm the presence and function of the *Ntv-a* construct *in vivo* (S1 Appendix, S1–S3 Figs). The initial phase of this study included injections of DF-1 cells transfected with RCAS-PDGFA and RCAS-FLuc constructs. The PDGFA only vector infection yielded neoplasia consistent with low-grade glioma approximately 250 days following injection (S4 and S5 Figs), similar to that observed in *Ntv-a* mice [7].

**Fig 2:**
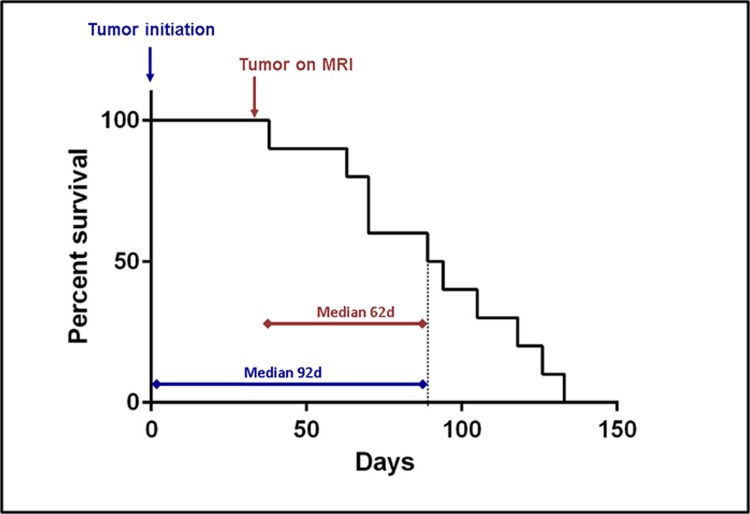
Glioma-bearing *Ntv-a* rat natural history study. Kaplan-Meir survival curve for PDGFA/p53 shRNA brain tumor-bearing animals. Median survival for PDGF-A/p53 shRNA tumor bearing animals from injection date was 92 [Interquartile Range [70–115]] days. Median survival from the first appearance of a tumor on the MRI was 62 days. All injected animals developed large malignant brain tumors.

**Fig 3:**
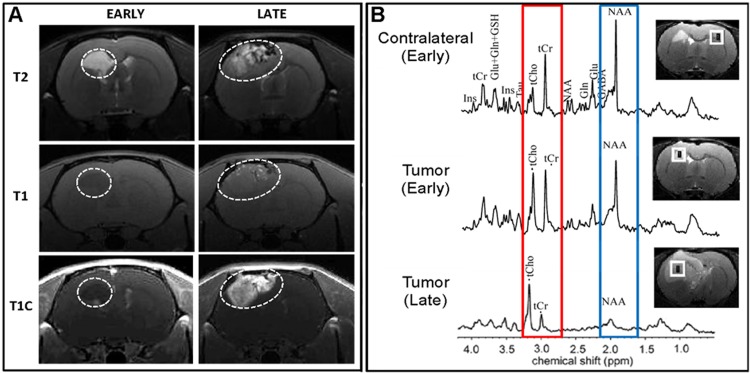
MRI and MRS analyses of brain tumor progression. **(A)** Early and late MRI time points using T2- and T1-weighted imaging. The early MRI findings are consistent with low-grade glioma, including T2 hyperintensity with discrete, homogeneous features. T1 imaging with (T1C) and without (T1) contrast enhancing dye shows little to no enhancement in the early timepoint image. The later imaging reveals a marked transformation in the same animal to a large, heterogeneous tumor with significant mass effect and midline shift, as well as early signs of obstructive hydrocephalus. This is confirmed by T1C imaging, which reveals marked and heterogeneous enhancement within the tumor. **(B)** The MRS comparing early and later brain tumor regions versus spectra from the contralateral cerebral hemispheres provides additional detail regarding malignant progression with evidence of increased cellular proliferation (red box: higher Cho/Cr) and expansion of non-neuronal tumor elements (blue box: decreased NAA).

To examine the effect of p53 depletion on tumorigenesis within the background of PDGFA overexpression, ten PND 10–14 *Ntv-a* rats underwent combined injections of DF-1 cells transfected with RCAS-PDGFA and RCAS-shp53 constructs. PDGFA overexpression and p53 depletion were confirmed by post-mortem brain tissue immunohistochemistry (S6 Fig). The median time to the experimental endpoint from tumor initiation was 92 days, and from tumor visualization with MRI, 62 days (Fig 2). The animals were monitored for signs of tumor progression through direct observation and MRI. Similar to the PDGFA alone-induced lesions, the initial tumors (day 30–40) with the PDGFA/shp53 combination showed MRI/S characteristics consistent with low-grade glial neoplasms (Fig 3). The tumors appeared homogenous with few to no signs of necrosis, angiogenesis, contrast enhancement or mass effect at this early stage (Fig 3A, left panels). Over time (~30 days), this pattern shifted to that of a high-grade glial tumor including marked mass effect, contrast enhancement and signal heterogeneity suggesting vascularity, necrosis, and brain invasion (Fig 3A, right panels). Spectroscopy revealed a shift in Cho/Cr and NAA peaks, consistent with the biological transformation toward malignancy (Fig 3B). Notably, all ten experimental animals in the combined RCAS-PDGFA/shp53 group developed large tumors with similar imaging and histological features, indicating 100% penetrance of glioma transformation in this model.

**Fig 4:**
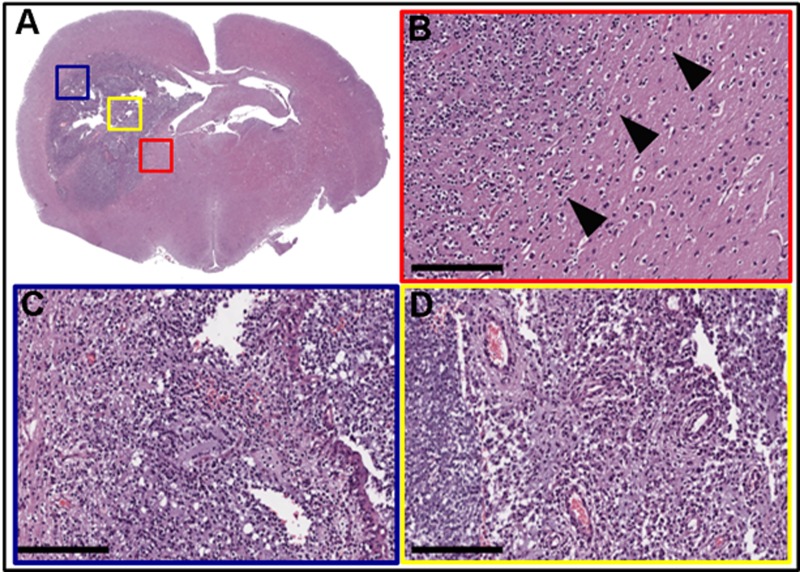
Histopathology of PDGFA/p53 shRNA-driven brain tumors. **(A)** Hematoxylin and eosin staining of whole brain slice with colored boxes highlighting areas of particular interest. (B) Red box = representative sample taken from the invasive edge of the tumor. Arrowheads indicate tumor invasion into adjacent brain tissue. **(C)** Blue box = hypercellular tumor with regions of pseudopallisading necrosis. **(D)** Yellow box = areas of microvascular proliferation, hypercellularity, and pleomorphism. (Scale bar = 200 μm).

**Fig 5:**
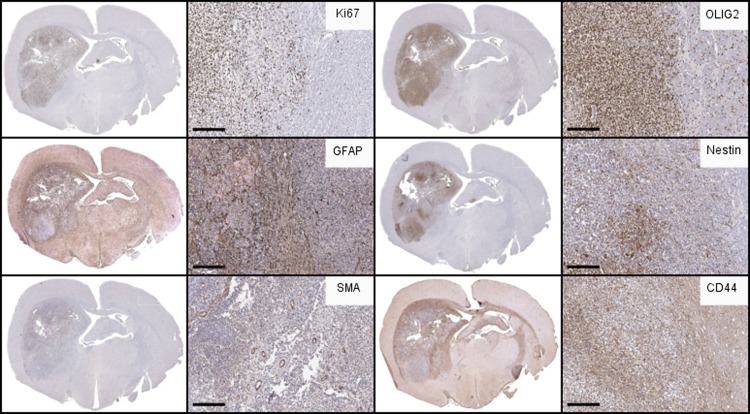
Immunohistochemical analysis of *Ntv-a* rat glioma. Immunohistochemical labeling of PDGFA/p53 shRNA-driven brain tumors reveals features in common with human GBM. Immunohistochemical (IHC) labeling (brown) for **(A)** Ki-67, demonstrating the high degree of proliferation in the tumor compared to the adjacent brain, and nests of proliferative cells in the invasive rim. **(B)** GFAP staining revealed variable staining with the greatest degree at the invasive tumor rim and regions of the tumor core. Notably, a significant portion of the tumor had minimal GFAP signal, reinforcing the proneural features of the tumor. **(C)** SMA (blood vessel marker) labeling detailed the extensive neo-vascularization within the tumor. **(D)** Olig2, considered a marker of glioma stemness and glial lineage, was strongly positive throughout the tumor. **(E)** Nestin labeling revealed persistent activation of neural stem-like progenitor cells throughout the tumor but not within the rest of the brain. **(F)** CD44 was broadly expressed through the brain pockets of strong positivity at the invasive areas near the tumor margins and associated white matter tracts. (Scale bar = 200 μm).

Post-mortem analysis revealed several key histological features of human glioblastoma, including cellular pleomorphism (Fig 4A), mitotic figures (Fig 4B), pseudopalisading necrosis (Fig 4C), and microvascular proliferation (Fig 4D). Tumor cells were visualized invading along white matter tracts (Fig 4A and 4B black arrowheads). Immunohistochemical analysis showed strong (>80% of cells) Ki-67 (Fig 5A) and variable GFAP immunolabeling (Fig 5B). This pattern is consistent with a high-grade, heterogeneous glial tumor.

Further immunolabeling was conducted to characterize molecular changes that occurred following gliomagenesis in tumor bearing animals (n≥3). SMA immunolabeling indicated extensive neo-vascularization within the tumor (Fig 5C). Olig2, a transcription factor that is highly expressed by gliomas [36], was observed within cells residing in both the tumor core and the invasive rim region (Fig 5D). Markers of tumor stem-like cells were also present in the tumors. Nestin staining revealed the presence of neural stem-like progenitor cells throughout the tumor but not within the rest of the brain (Fig 5E). Interestingly, CD44, a previously characterized marker of tumor stem-like cells [37, 38] and highly expressed in MES gliomas [39], was broadly expressed throughout the brain with specifically strong positivity in the perivascular spaces and the invasive regions near the tumor margins and associated white matter tracts (Fig 5F).

**Fig 6:**
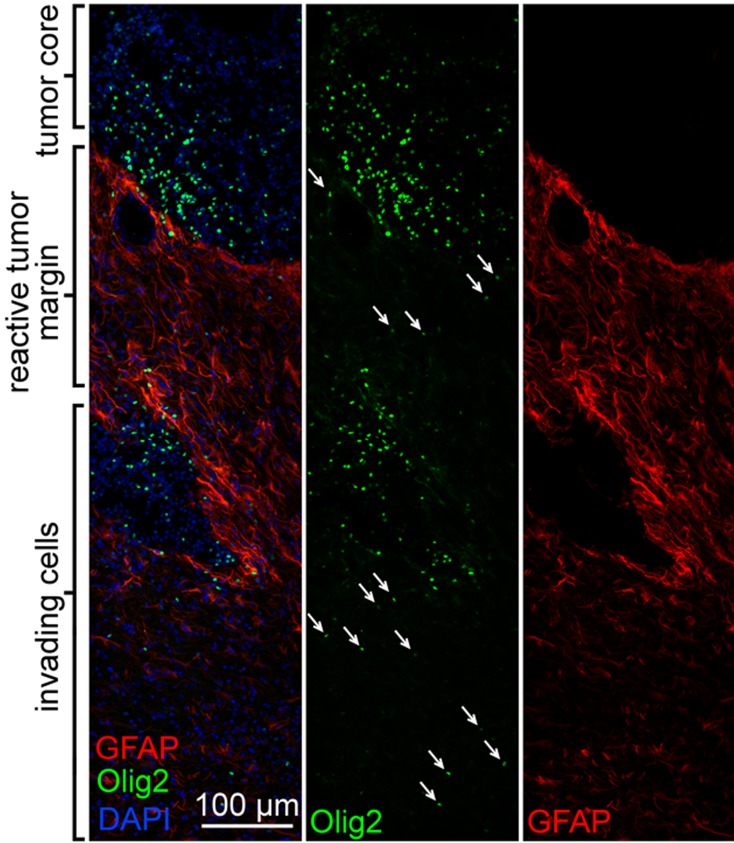
Immunofluorescent labeling of brain tumors reveals brain invasion and reactive processes at the tumor margin. Oligodendroglial tumor cells (Olig2+) invading through and within the reactive astrocytosis (GFAP+) in the tumor margin.

**Fig 7:**
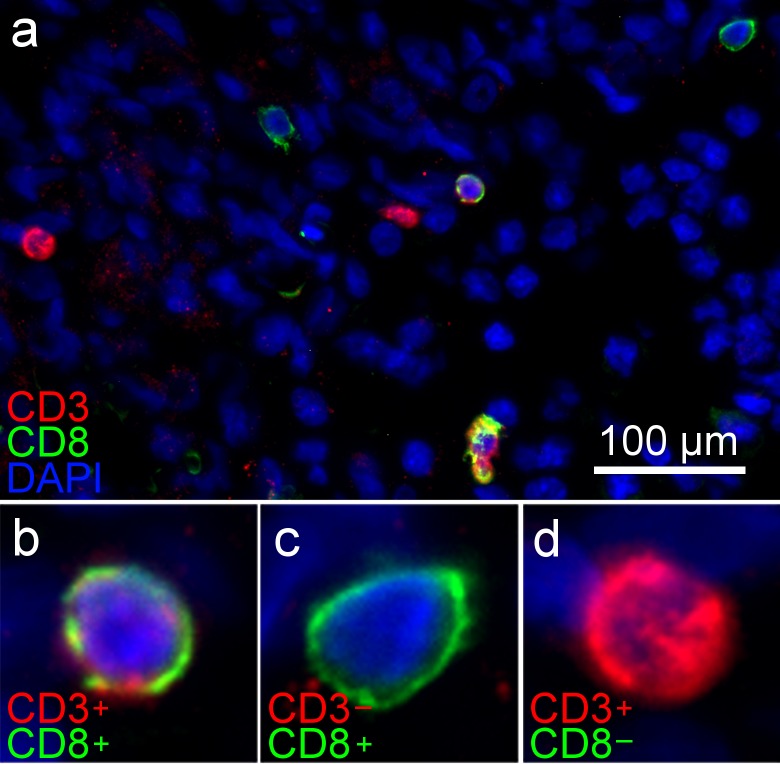
Immunofluorescent labeling of brain tumors shows infiltration of lymphocytes of varying immunophenotypes. **(A)** Sparse lymphocyte infiltration within the tumors is demonstrated by CD3 and CD8 co-labeling revealing **(B)** CD3+/CD8+ cells suggesting cytotoxic T-cells, **(C)** CD3+/CD8- cells reflecting helper and/or regulatory T-cells, and **(D)** CD3-/CD8+ cells, possibly NK or dendritic cells.

**Fig 8:**
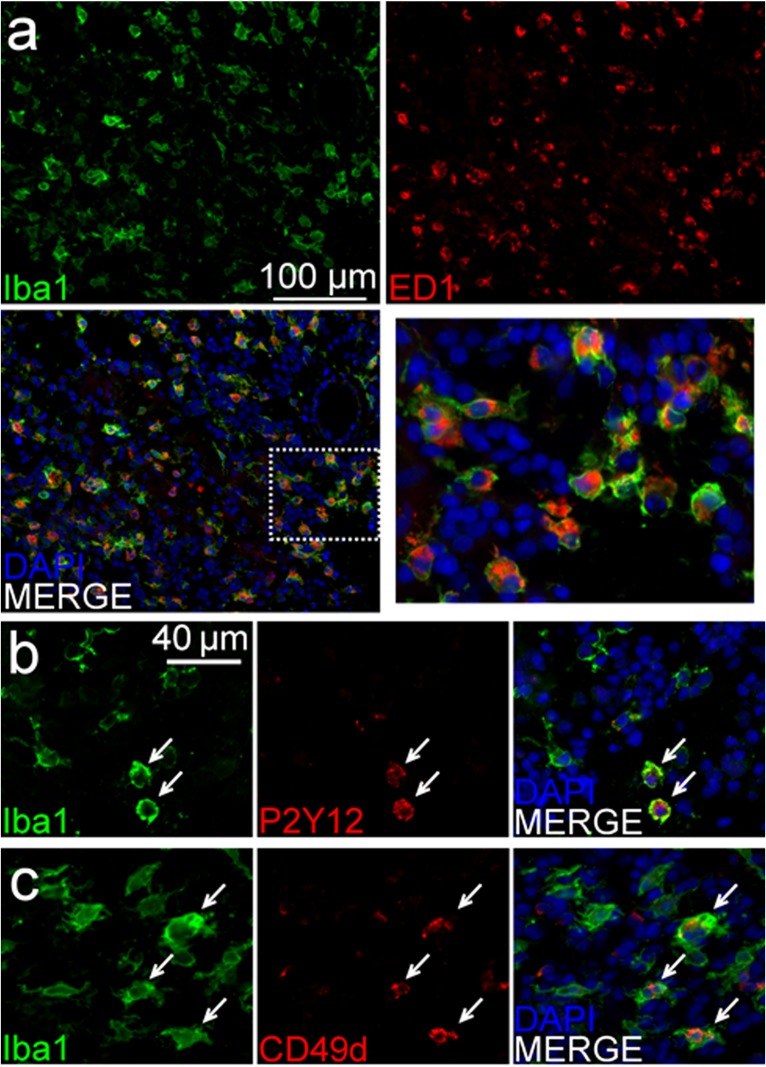
Immunofluorescent labeling of brain tumors reveals dense and diverse monocytic tumor infiltration. **(A)** Labeling of tumor-associated macrophages and microglia (Iba-1+) showed dense infiltration within the tumor and a high degree of cellular activation (ED-1+). **(B)** Further delineation of microglia (P2Y12+)- and **(C)** bone marrow-derived monocytes (CD49d+) revealed separate contributions from each of these unique cell populations and lineages to the tumor ecosystem. Of note, co-labeling with Iba-1 and each P2Y12 and CD49d showed some overlap (white arrows) but also some distinct, Iba-1 only positive cells.

**Fig 9:**
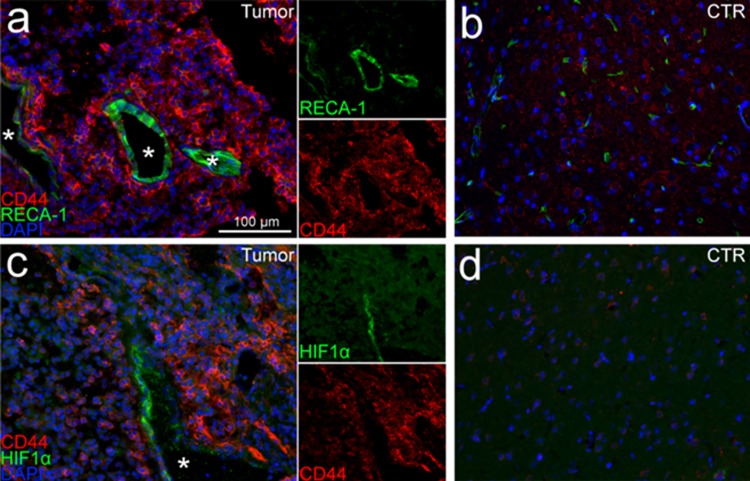
Immunofluorescent labeling of brain tumors reveals microvascular proliferation and perivascular stem-like cell niche formation. **(A)** Dense populations of stem-like cells (CD44+) around large, healthy neovascular structures (RECA-1+) were visualized within the tumors (white stars represent vessel lumens). **(B)** The contralateral cerebral hemisphere (control (CTR)) showed low-level CD44 positively and fewer small blood vessels. **(C)** The neovascular tumor regions also showed perivascular pockets of HIF-1+ cells, a factor linked to tumor angiogenesis and other oncogenic adaptations [45] (white star represents vessel lumen). **(D)** Brain tissue in the contralateral hemisphere showed no evidence of HIF-1+ cells.

Immunofluorescent microscopy was used to better characterize the inter-relationships between the *de novo* formed tumors and the rat brain microenvironment. Olig2 and GFAP co-labeling demonstrated numerous glial tumor cells (OLIG2+) invading through and within a peri-tumoral band of reactive astrocytes (GFAP+) in the tumor margin (Fig 6). Labeling of lymphocytes (Fig 7A) showed a pattern suggesting a sparse infiltration of a diverse population of lymphocytes within the tumors, including evidence of possible cytotoxic T-cells (CD3+/CD8+) (Fig 7B), helper and/or regulatory T-cells (CD3+/CD8-) (Fig 7C), and natural killer (NK) or dendritic cells (CD3-/CD8+) (Fig 7D). Labeling of macrophages and microglia (Iba1+) showed dense infiltration of numerous activated macrophages (ED1+) [40, 41] into the tumor core (Fig 8A). Further delineation of microglia (P2Y12+) [42, 43] and bone marrow-derived macrophages (CD49d+) [44] revealed separate contributions from each of these unique cell populations and lineages to the tumor ecosystem (Fig 8B). Lastly, CD44 and RECA-1 co-labeling demonstrated numerous foci of stem-like cells (CD44+) that were clustered around large atypical neovascular structures lined with endothelial cells (RECA-1+) (Fig 9A; contralateral tissue Fig 9B). Regions containing foci of CD44+ cells exhibited HIF-1α immunolabeling (Fig 9C; contralateral tissue Fig 9D), a transcription factor linked to tumor angiogenesis and other oncogenic adaptions [38, 45].

## Discussion

We investigated glioma initiation and progression driven by PDGFA/p53-/-in *tv-a* transgenic rats. This model permits cell-type, temporal, and spatial control of oncogenic gene transformations based on the RCAS/*tv-a* system. Tumor initiation was studied in *nestin*-positive neural and glial progenitor cells located in the frontal, periventricular region—the cells implicated in the formation of and a common location for human GBM. PDGFA, frequently overexpressed in the proneural GBM molecular subtype and considered to be a key initiating alteration in gliomagenesis, was introduced alone and in combination with a construct knocking down the p53 tumor suppressor that is mutant in nearly all astrocytomas and a subset of GBM [4, 5]. The combination of PDGFA overexpression and p53 depletion resulted in consistent high-grade tumor formation. These tumors initially resembled low-grade neoplasms and then progressed to large, heterogeneous tumors in all experimental animals. This biological process and pattern were visualized by MRI and MRS—methodologies similar to clinical protocols. Histopathological analysis showed many of the classic features of human GBM including necrosis, microvascular proliferation, cellular pleomorphism, high proliferation rates, and invasion along white matter tracts, as well as strong OLIG2, CD44, and nestin staining with mixed GFAP staining. Immunofluorescent co-labeling further highlighted the invasive and highly reactive elements of these glial neoplasms including peripheral astrocytosis and marked infiltration of a diverse population of inflammatory cells within the tumor, including lymphocytes and both microglia- and bone marrow-derived macrophages. HIF-1+ tumor regions with robust microvascular proliferation were also visualized, as well as associated stem-like elements suggesting recreation of the human GBM feature described as the ‘perivascular niche’ [38]. Taken together, this inducible rat transgenic glioma model offers highly-relevant histology, MR imaging, and biological inter-relationships related to accurate modeling of formation and progression in human glioma.

The RCAS/*tv-a* technology, originally developed to model brain cancer in mice [18], has enabled numerous important observations related to gliomagenesis and pathobiology, as well as served as the foundation for multiple diagnostic and therapeutic studies [7, 15, 18, 21, 22, 27, 46, 47]. In the original study, combined activation of the Ras and Akt oncogenes in neural progenitor cells was found to generate high-grade gliomas in mice. Importantly, the tumors formed *de novo* and appeared to arise only after gene transfer to neural progenitor (*nestin* positive) cells and not differentiated astrocytes [18]. In subsequent work, the *tv-a* mouse model was used to explore the evolution of the most common form of primary GBM, those tumors with a wild-type isocitrate dehydrogenase (IDH) gene, also described as the non ‘glioma-CpG island methylator phenotype’ (GCIMP) [7]. In this study, the investigators observed that most non-GCIMP tumors formed from a common proneural-like precursor driven in large part by alterations in PDGF expression [7]. These findings were validated through gene set analysis with data from GBM patients in the TCGA database. In addition, the tumor-immune microenvironment that develops in RCAS/*tv-a* -derived tumors has been shown to be similar to that found in humans, especially compared to another commonly used cell transplant model in immunocompetent mice—the GL261 glioblastoma cell model [48]. Indeed, the present study revealed numerous key similarities between the tumors formed in *Ntv-a* rats and humans, in particular the reactive and infiltrating cellular processes, and perivascular stem cell niche [7, 48]. The correlation between RCAS/*tv-a* brain tumors and human GBM has led to the utilization of this model in molecular imaging studies [46], immunosuppression and mechanisms of tumor progression investigations [20, 49], and studies examining new therapeutics [21, 50].

The emerging understanding of GBM disease mechanisms reveals the importance of tumor size, heterogeneity, immunologic interplay, and gene expression signatures in modeling this complex disease. Patel and colleagues determined the RNA sequencing profiles of individual cells in patient GBM samples [6]. They found variable and diverse transcriptional programs related to oncogenic signaling, proliferation, and immune responses. They also examined the individual GBM molecular subtypes in these cells and found significant variability. This information suggests that regional tumor characteristics are likely to be important in accurately modeling the intratumoral heterogeneity in GBM. The findings in this study reveal *de novo* formed, heterogeneous tumors with diverse inflammatory components.

Detailed descriptions of differences in physiology and anatomic structure between mice, rats, and other mammalian host species, reveal opportunities to model specific diseases, biological processes, and treatments in the background environment that most closely recreates the human disease [51–55]. Rat models have been widely used to study GBM and evaluate numerous treatments, including interstitial and local therapies, fractionated and targeted radiation, and surgical resection-based approaches. These studies have included non-specific chemical- or virus-induced syngeneic versions (e.g. 9L, C6, F98, CNS-1 cells) [10, 56] human xenografts grown in immuno-deficient host rats [57], and genetically engineered spontaneous rat tumors [58, 59]. Historically, pre-clinical testing in rat model(s) has been a critical step in the pathway to FDA-approval [60–63]. Rat models offer some distinct advantages and disadvantages compared to mouse models of human disease. Compared to other disease host species, rats have relatively complex and modifiable behaviors as well as numerous differences in immune function [64–67]. In addition, the size of rat brain offers the opportunity for close stereotactic localization of tumor initiation site to study differences related to brain location. It is possible that the rat brain size will allow a longer time interval from tumor initiation to death, which often occurs due to mass effect and neurological dysfunction. This in turn may permit more time for GBM disease elements to be recreated, including intratumoral heterogeneity, distant brain invasion, and immune evasion. Regarding treatments, rats broaden the pre-clinical application of delivery modalities like convection enhanced delivery, MR-guided focused ultrasound, and interstitial implantation.

Some relative disadvantages of rat models include higher caging and husbandry costs, and relatively fewer commercially available research reagents (e.g. monoclonal antibodies and cytokine assays). Despite the historical value of rats in neuroscience and neuro-oncology research, rat brain tumor models have not been available for temporal and spatially controlled gene transformations in neural precursor cells to study glioma formation, progression, and treatments. The development of this *Ntv-a* transgenic rat model, as well as the recent expansion of rat-related resources for cancer and other research [68, 69], offer new opportunities for the use of rats in experimental and translational neuro-oncology, as well as broader neuroscience applications. It is likely that in the near future experimental brain tumor models will be characterized based on their accuracy in recreating specific human disease elements and processes, which may closely relate to the background features of the host species. We plan to utilize this model to further characterize differences in intratumoral molecular heterogeneity and gene expression across various model species and correlate these findings with emerging information from human studies.

In conclusion, an *Ntv-a* transgenic rat colony was established and used to study glial brain tumors driven by two well-characterized oncogenic gene transformations, PDGFA overexpression and p53 depletion. Combined PDGFA and p53 alterations resulted in large, heterogeneous tumors in all affected animals. Histopathological and MRI examinations showed many of the key features observed in human proneural GBM. The close inter-relationships between tumor cell invasion and the reactive cells within the brain (astrocytes, microglia) and those infiltrating from outside the brain (bone marrow-derived monocytes) were demonstrated, as well as key features of glioma blood vessels and the perivascular stem-like cell niche. The collection of RCAS vectors created for the mouse system can be used with these *tv-a* transgenic rats, and the knowledge gained in the mouse experiments can guide the creation of genetically tailored gliomas in the rat.

## Supporting information

S1 Appendix Supplemental methods and results. This file contains supplementary methods and results regarding the creation of the model and further confirmation of the model’s effectiveness.
(DOCX)

S1 Fig Transgene copy number analysis. Cq values of serial dilution (log 2.5 to log 5.5) of nestin plasmid DNA. Standard curve equation y = -3.317x+46.644 (R^2^ = 0.999) serves as basis for sample DNA copy number determination.
(TIF)

S2 Fig *tv-a* immunohistochemistry on rat brains. **(A)** IHC using a *tv-a* monoclonal antibody showed diffuse positivity in the PND10 Ntv-a rat brain compared to **(B)** minimal staining in adult PND60 *Ntv-a* rat brain. **(C)** Negative isotype control staining of PND10 *Ntv-a* rat brain. (Scale bar = 200 μm).
(TIF)

S3 Fig Verification of *Ntv-a* expression and function following injection of DF-1 RCAS-FLuc and PDGF-A cells into N*tv-a* transgenic rats. Bioluminescence imaging was performed using standard techniques at the indicated time points.
(TIF)

S4 Fig Brain tumor formation following PDGF-A transformation alone: MRI and MR spectroscopy. **(A)** Coronal and **(B)** axial MRI of animal at 250 days post-injection. The MRI reveals findings consistent with low-grade glioma, including T2 hyperintensity with discrete, homogeneous features. **(C)** The MR spectroscopy provides additional detail regarding tumor characteristics compared to a similar region within the contralateral cerebral hemisphere, including evidence of mildly elevated cellular proliferation (red box: moderately increased Cho/Cr) and some expansion of non-neuronal tumor elements (blue box: decreased NAA).
(TIF)

S5 Fig Histopathology of PDGF-A-driven brain tumor. **(A)** H&E staining of brain section shows a moderately cellular tumor with relatively **(B)** discrete borders and **(C)** minimal microvascular proliferation or necrosis. Immunohistochemistry revealed **(D)** Ki-67 staining in ~10–20% of the tumor cells, and **(E)** strong OLIG2 staining including diffuse positivity throughout the ipsilateral hemisphere and white matter tracts. **(F)** GFAP staining was mixed and appeared in regions of blood vessels. (Scale bar = 200 μm).
(TIF)

S6 Fig Immunohistochemical staining for PDGF-A and mCherry expression. Strong positive and negative staining of **(A)** PDGF-A and **(B)** p53, respectively, was observed in the PDGF-A/p53 shRNA-generated brain tumors, confirming these gene transformations in the tumor tissue. **(C)** IHC of mCherry tag included on the p53 shRNA construct shows positive staining within tumor further confirming gene transformation. (Scale bar = 400 μm).
(TIF)
