# Supplementary material for: Genetically engineered rat gliomas: PDGF-driven tumor initiation and progression in tv-a transgenic rats recreate key features of human brain cancer
Source: PLoS One. 2017 Mar 30;12(3):e0174557. doi: 10.1371/journal.pone.0174557 (PMC5373526; doi:10.1371/journal.pone.0174557)
Supplement: S1 Appendix — This file contains supplementary methods and results regarding the creation of the model and further confirmation of the model’s effectiveness. (DOCX) [file pone.0174557.s001.docx]

# **Supplemental Results**

## **Establishment of Ntv-a transgenic rat colony**

Founder lines were genotyped for the *tv-a* gene and all *tv-a*-negative animals were excluded from the study. Breeding pairs were set up among a selected founder line to create the first offspring group. This group was 90% positive for the *tv-a* gene. The copy number of each animal was determined using a serial dilution of the nestin plasmid (Transposagen) ranging from 300 to 300,000 copies (Fig S1). Using the resulting Cq values, a linear equation was determined (y = (-3.55) x + 16.34; R^2^=0.99) and would provide the basis for log copy number determination for subsequent offspring (GraphPad Prism version 6.00 for Windows, GraphPad Software, La Jolla, CA)**.** The first set of offspring (F1), displayed a log copy number value ranging from 4.21 to 6.79 log copies. The male with the highest copy number (6.79) and the female with the highest copy number (6.67) of the F1 group were bred to create a second offspring (F2) group which presented with a range of 5.72 to 6.75 log copy number. Once again, the highest male (6.67) and female (6.5) of F2 were bred and yielded the offspring (F3) that are presented in this study. The animals of F3 that developed tumors displayed log copy numbers ranging from 6.5 to 7.04. The copy number determination allowed the establishment and maintenance of a high copy number breeding schema and animal colony. High copy offspring were used for the tumor initiation studies**.** IHC using brain tissue confirmed transgene integration and expression of the avian *tv-a* receptor in these offspring (Fig S2).

## **Initial RCAS FLuc and PDGF-A co-injection experiments into transgenic rat brain confirmed Ntv-a expression and progenitor cell transformation**

To confirm the function of the *Ntv-a* transgene *in vivo*, DF-1 cells producing RCAS-FLuc or RCAS-PDGF-A virions were co-injected into PND10 rats. A BLI signal was visible in the brain for seven days (Fig S3). These rats were monitored over time through direct observation. One rat was examined on post-injection day 250 by MRI and a small T2 hyper-intense lesion was observed. Comparing the spectroscopic pattern to a similar brain region in the contralateral cerebral hemisphere, the lesion had characteristics consistent with a low grade glial neoplasm including increasing choline to creatinine ratio and decreasing NAA [15, 41] (Fig S4). Histopathological analysis showed a moderately cellular tumor with relatively discrete borders and minimal microvascular proliferation or necrosis (Fig S5A-C). Immunohistochemistry revealed Ki-67 positivity (Fig S5D) and strong OLIG2 staining including diffuse signal throughout the ipsilateral hemisphere and white matter tracts (Fig S5E). GFAP staining was mixed and appeared in regions of blood vessels (Fig S5F). Taken together, these findings are consistent with formation of a PDGF-A-driven low-grade oligodendroglial-lineage neoplasm.
