# Supplementary figures and images for: Genetically engineered rat gliomas: PDGF-driven tumor initiation and progression in tv-a transgenic rats recreate key features of human brain cancer
Source: PLoS One. 2017 Mar 30;12(3):e0174557. doi: 10.1371/journal.pone.0174557 (PMC5373526; doi:10.1371/journal.pone.0174557)

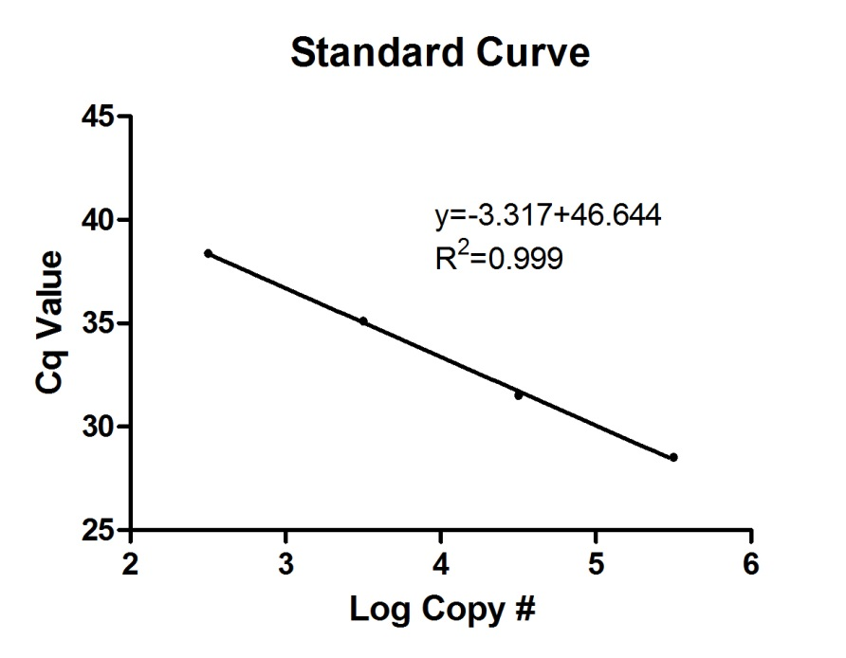

Supplement: S1 Fig — Standard curve equation y = -3.317x+46.644 (R2 = 0.999) serves as basis for sample DNA copy number determination. (TIF) [file pone.0174557.s002.tif]

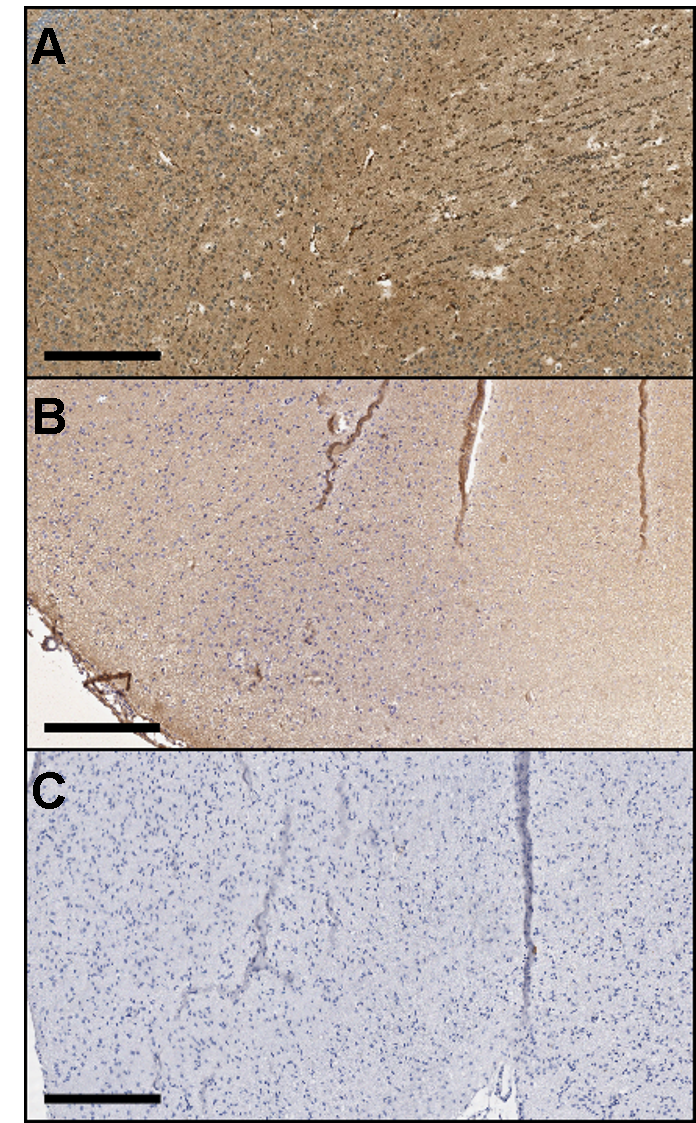

Supplement: S2 Fig — (A) IHC using a tv-a monoclonal antibody showed diffuse positivity in the PND10 Ntv-a rat brain compared to (B) minimal staining in adult PND60 Ntv-a rat brain. (C) Negative isotype control staining of PND10 Ntv-a rat brain. (Scale bar = 200 μm). (TIF) [file pone.0174557.s003.tif]

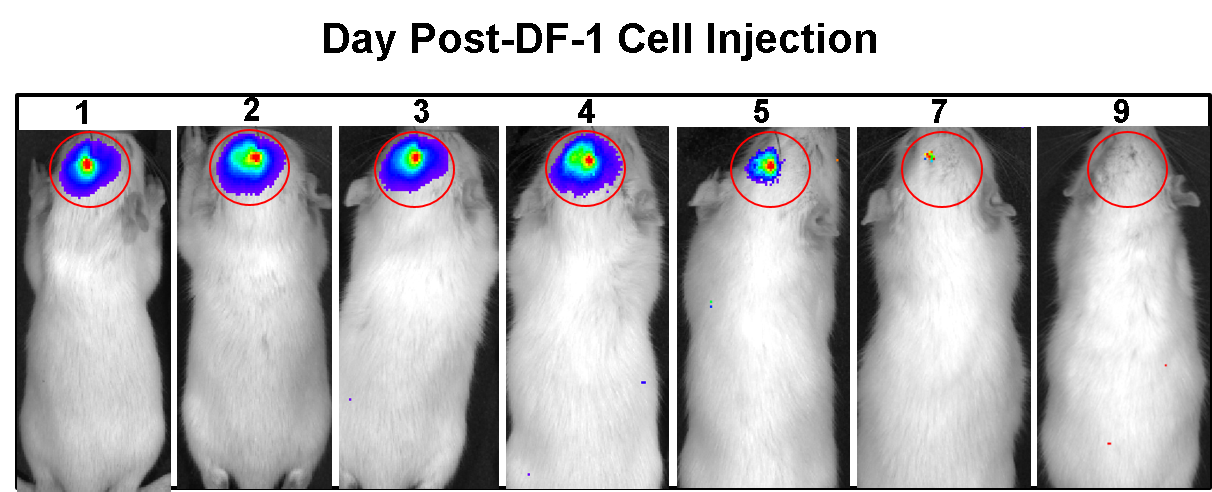

Supplement: S3 Fig — Bioluminescence imaging was performed using standard techniques at the indicated time points. (TIF) [file pone.0174557.s004.tif]

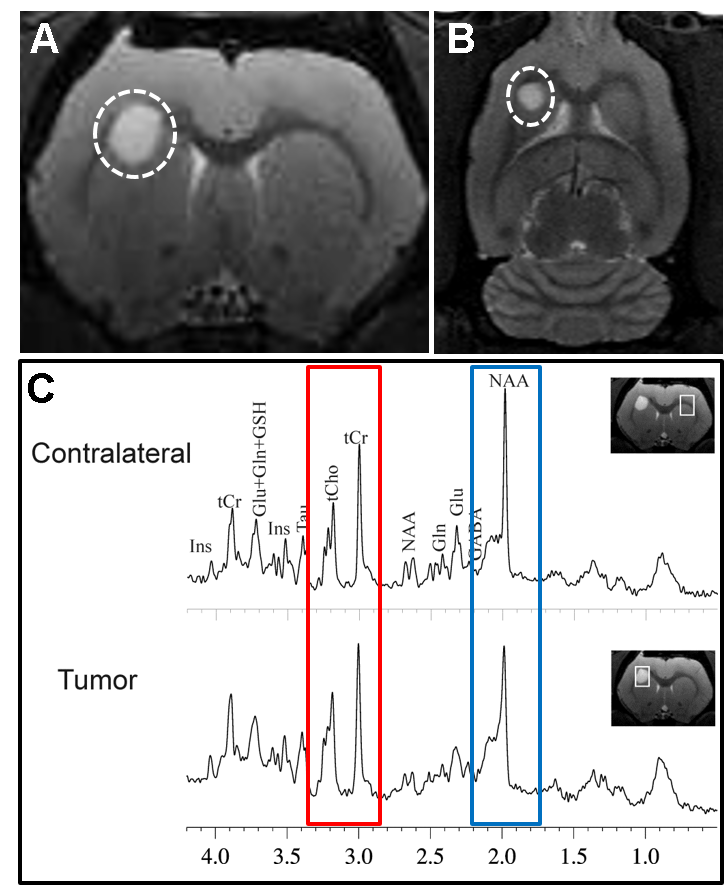

Supplement: S4 Fig — (A) Coronal and (B) axial MRI of animal at 250 days post-injection. The MRI reveals findings consistent with low-grade glioma, including T2 hyperintensity with discrete, homogeneous features. (C) The MR spectroscopy provides additional detail regarding tumor characteristics compared to a similar region within the contralateral cerebral hemisphere, including evidence of mildly elevated cellular proliferation (red box: moderately increased Cho/Cr) and some expansion of non-neuronal tumor elements (blue box: decreased NAA). (TIF) [file pone.0174557.s005.tif]

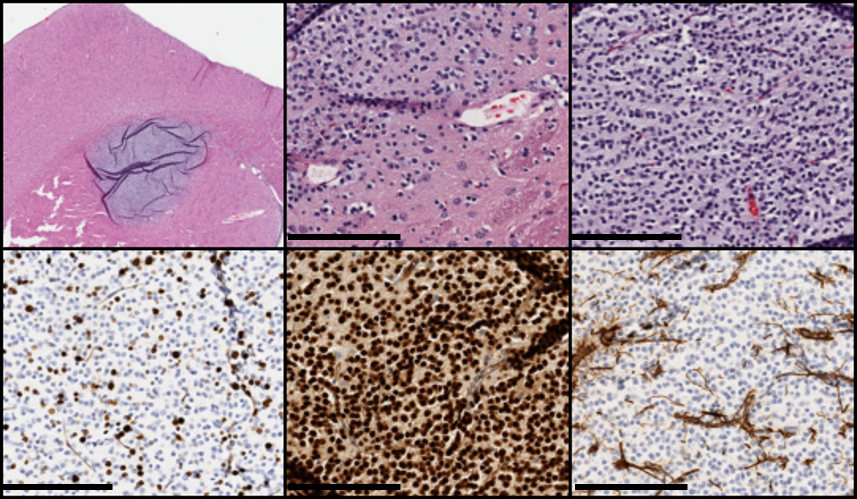

Supplement: S5 Fig — (A) H&E staining of brain section shows a moderately cellular tumor with relatively (B) discrete borders and (C) minimal microvascular proliferation or necrosis. Immunohistochemistry revealed (D) Ki-67 staining in ~10–20% of the tumor cells, and (E) strong OLIG2 staining including diffuse positivity throughout the ipsilateral hemisphere and white matter tracts. (F) GFAP staining was mixed and appeared in regions of blood vessels. (Scale bar = 200 μm). (TIF) [file pone.0174557.s006.tif]

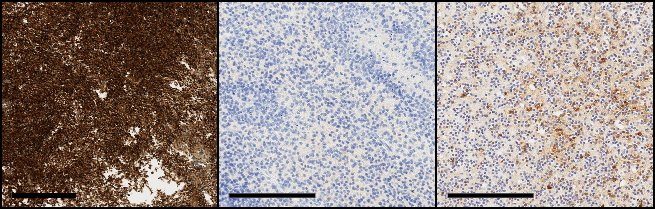

Supplement: S6 Fig — Strong positive and negative staining of (A) PDGF-A and (B) p53, respectively, was observed in the PDGF-A/p53 shRNA-generated brain tumors, confirming these gene transformations in the tumor tissue. (C) IHC of mCherry tag included on the p53 shRNA construct shows positive staining within tumor further confirming gene transformation. (Scale bar = 400 μm). (TIF) [file pone.0174557.s007.tif]
